# Do Natural T Regulatory Cells become Activated to Antigen Specific T Regulatory Cells in Transplantation and in Autoimmunity?

**DOI:** 10.3389/fimmu.2013.00208

**Published:** 2013-08-02

**Authors:** Bruce M. Hall, Giang T. Tran, Nirupama D. Verma, Karren M. Plain, Catherine M. Robinson, Masaru Nomura, Suzanne J. Hodgkinson

**Affiliations:** ^1^Immune Tolerance Laboratory, Medicine, University of New South Wales, Sydney, NSW, Australia

**Keywords:** antigen specific T_reg_, nT_reg_, Th1-like T_reg_, Th2-like T_reg_, immune tolerance

## Abstract

Antigen specific T regulatory cells (T_reg_) are often CD4^+^CD25^+^FoxP3^+^ T cells, with a phenotype similar to natural T_reg_ (nT_reg_). It is assumed that nT_reg_ cannot develop into an antigen specific T_reg_ as repeated culture with IL-2 and a specific antigen does not increase the capacity or potency of nT_reg_ to promote immune tolerance or suppress *in vitro*. This has led to an assumption that antigen specific T_reg_ mainly develop from CD4^+^CD25^−^FoxP3^−^ T cells, by activation with antigen and TGF-β in the absence of inflammatory cytokines such as IL-6 and IL-1β. Our studies on antigen specific CD4^+^CD25^+^ T cells from animals with tolerance to an allograft, identified that the antigen specific and T_reg_ are dividing, and need continuous stimulation with specific antigen T cell derived cytokines. We identified that a variety of cytokines, especially IL-5 and IFN-γ but not IL-2 or IL-4 promoted survival of antigen specific CD4^+^CD25^+^FoxP3^+^ T_reg_. To examine if nT_reg_ could be activated to antigen specific T_reg_, we activated nT_reg_ in culture with either IL-2 or IL-4. Within 3 days, antigen specific T_reg_ are activated and there is induction of new cytokine receptors on these cells. Specifically nT_reg_ activated by IL-2 and antigen express the interferon-γ receptor (IFNGR) and IL-12p70 (IL-12Rβ2) receptor but not the IL-5 receptor (IL-5Rα). These cells were responsive to IFN-γ or IL-12p70. nT_reg_ activated by IL-4 and alloantigen express IL-5Rα not IFNGR or IL-12p70Rβ2 and become responsive to IL-5. These early activated antigen specific T_reg_, were respectively named Ts1 and Ts2 cells, as they depend on Th1 or Th2 responses. Further culture of Ts1 cells with IL-12p70 induced Th1-like T_reg_, expressing IFN-γ, and T-bet as well as FoxP3. Our studies suggest that activation of nT_reg_ with Th1 or Th2 responses induced separate lineages of antigen specific T_reg_, that are dependent on late Th1 and Th2 cytokines, not the early cytokines IL-2 and IL-4.

## Historical Perspective

Immune tolerance results from a combination of deletion of antigen specific T and B cell clones, anergy, and suppression. Like all biological systems, immunity has in built self-regulation that prevents induction of destructive autoimmunity and controls or limits all immune effector responses against any antigen. While a variety of leukocytes can regulate, this review will focus only on CD4^+^ T regulatory cells (T_reg_).

Since the first description of suppressor T cells, the difference between non-antigen specific T_reg_ that reside in thymus, bone marrow, and peripheral lymphoid tissues, and antigen specific T_reg_ that are present mainly in spleen and tissues, has been appreciated (1–3). This division is consistent with natural T_reg_ (nT_reg_) and antigen specific T_reg_. Early studies characterized CD8^+^ T suppressor cells, reviewed (4) but this work was discredited (5) and a common view was suppressor T cells did not exist, until the recognition of CD4^+^ T_reg_.

### Antigen specific CD4^+^CD25^+^ T_reg_

Alloantigen specific transplant tolerance was found in the mid 1980s to be mediated by CD4^+^ T cells not CD8^+^ T or B cells (6–8). In the early 1990s Waldman’s group found CD4^+^ T cells from host transplant tolerant animals infect adoptive hosts’ T cells to maintain alloantigen specific tolerance (9).

At that time, we observed that the CD4^+^ T cells that transferred antigen specific tolerance rapidly died *in vitro* (10–12). Death of antigen specific tolerance transferring CD4^+^ T cells could be prevented by both stimulation with specific antigen and cytokines provided at that time by supernatant from Concanavalin A stimulated spleen cells. This supernatant was a crude source of IL-2 (12), but is now known to contain a number of cytokines, as well as IL-2. This suggested that the CD4^+^ T cells that transfer transplant tolerance were activated cells that may depend on IL-2. We thus examined and found they expressed the IL-2 alpha receptor (CD25) (11). In 1990 we identified alloantigen specific tolerance transferring cells as CD25^+^ Class II MHC^+^CD45RC^+^CD4^+^ T cells (11). At that time CD25 was expressed by CD4^+^ T cells activated to effect rejection (13), thus we assumed the suppressor cells were derived from specific alloantigen activated CD4^+^ T cells. As IL-2 alone only partially sustained the capacity of tolerant CD4^+^ T cells to transfer antigen specific tolerance, we concluded other cytokines were required (12). Since we have systematically examined which cytokines are involved in the maintenance of antigen specific CD4^+^CD25^+^FoxP3^+^ T_reg_, and this is the focus of this review.

### Natural T_reg_

We also found that normal animals have cells, particularly in thymus and bone marrow, that suppress immune responses in a non-antigen specific manner, and that adult thymectomy depletes these cells, leading to heightened immune responses (14) and greater susceptibility to autoimmunity (15). Alloantigen specific CD4^+^ T suppressor cells have a different tissue distribution, being greatest in spleen, less in lymph nodes, and not in thymus or bone marrow (7). Further, they do not re-circulate rapidly from blood to lymph, suggesting they re-circulated through peripheral somatic tissue not through lymphoid tissues (7), similar to memory T cells (16), and not like naïve T cells that re-circulate from blood through lymphoid tissues (17). These basic differences in the migration of antigen specific and nT_reg_ can be used to distinguish these cell populations by cell surface markers that direct their migration pathways, reviewed (18).

Later, activated CD4^+^ T cell in normal animals that expressed CD25 and prevented autoimmunity in neonatal thymectomized mice were described (19). These CD4^+^CD25^+^ T_reg_ suppressed in a non-antigen specific manner, and are known as nT_reg_. nT_reg_ are thymus derived and express FoxP3 (20) that prevents IL-2 induction and induces CD25 expression. FoxP3 expression in mice is a marker of T_reg_, but in man activated CD4^+^ and CD8^+^ T cells transiently express FoxP3 (21) and can be induced to have prolonged expression of FoxP3 (22). IL-2 is essential for survival of nT_reg_ in peripheral lymphoid tissues (23, 24). CD4^+^ T cell with high expression of CD25, are regulatory, whereas CD4^+^CD25^lo^ T cells are not regulatory (25).

Natural T_reg_ have low expression of CD127, the IL-7 receptor, which is highly expressed by effector lineage CD4^+^CD25^−^ T cells (26), albeit activated CD4^+^ T cells (27), and T follicular helper cells (Tfh) also have low expression of CD127 (28). The survival of nT_reg_ without an immune response is dependent on low levels of IL-2, whereas CD4^+^CD25^−^ T cells depend upon IL-7 (29) not IL-2 for their survival without antigen activation. In the thymus IL-2 (30), not IL-7 (31) is critical for production of nT_reg_, although IL-7 plays a separate role in induction of nT_reg_ in the thymus (32).

The CD4^+^CD25^+^FoxP3^+^ T cells are a heterogeneous group, and include naïve nT_reg_ produced by the thymus, that have TCRs with increased affinity for self either due to thymic selection for self or expansion of self reactive clones in the periphery (33, 34). These naïve nT_reg_ are polyclonal, with a wide repertoire of TCR. In normal immunological naïve hosts, some naïve nT_reg_, with TCR specific for autoantigens, may have contacted antigen and been activated or expanded, to increase the repertoire of autoreactive nT_reg_. In addition, especially in hosts with acquired immune tolerance, there may be CD4^+^CD25^+^ T_reg_ reactive to foreign or alloantigens, that have been expanded and function as antigen specific T_reg_. These are no longer naïve nT_reg_. Hosts with established antigen specific tolerance may have a large population of activated T_reg_ with TCR specific for the tolerated antigen that mediate this tolerance, as well as the normal naïve nT_reg_ with a TCR repertoire for self as well as a limited repertoire for other foreign antigens.

### Induction of T_reg_ from CD4^+^CD25^−^ T cells

CD4^+^CD25^−^ T cells can be activated by antigen in the absence of inflammatory cytokines, to antigen specific T_reg_. The first induced T_reg_ (iT_reg_) described by Weiner are Th3 cells induced by TGF-β in oral tolerance, reviewed (35). Groux et al. described induction of antigen specific T_reg_ by repeated culture of CD4^+^ T cells with antigen and IL-10, producing Tr1 cells that suppress via production of IL-10 and TGF-β (36). Tr1 and Th3 cell do not express CD25 or FoxP3 (35, 37).

Induced T_reg_ are derived from peripheral CD4^+^ T cells that are stimulated by antigen and TGF-β in the absence of inflammation and inflammatory cytokines. These iT_reg_ are induced to express FoxP3, albeit its expression is not stable as the T_reg_ specific demethylation region (TSDR or CBS2) for FoxP3 is not demethylated (38). Both TGF-β which down regulates many genes, and FoxP3 expression which down regulates other genes, are required to induce iT_reg_ from CD4^+^ T cells (39).

Most attempts to describe T_reg_ oversimplify the complex nature of these cells *in vivo*, by describing all T_reg_ as one type of cells, or dividing their description into nT_reg_ and iT_reg_. nT_reg_ remain non-antigen specific polyclonal T_reg_ when cultured with IL-2 alone, whereas antigen specific nT_reg_ are not expanded by IL-2. This and the small frequency of nT_reg_ reactive to a specific antigen has led some to conclude that some, if not the majority, of antigen specific T_reg_ reactive to foreign antigens may be derived from iT_reg_ and not from activation of nT_reg_ (40–43). The lack of a distinct surface marker to distinguish antigen specific T_reg_ produced as iT_reg_ from those derived from nT_reg_, makes determination of the precise contribution of nT_reg_ and iT_reg_ to states of induced tolerance difficult (44, 45).

This review will focus on antigen specific T_reg_ induced from nT_reg_, not on iT_reg_. Most of the material presented is derived from murine models. In each section, murine results will be presented first, then any human data will be discussed. At the end of each section, any information on similar cells derived from iT_reg_ will be briefly mentioned.

Our work on T_reg_ has shown that differential cytokine receptor expression is key to the identification of different T cell subtypes, including nT_reg_ (46). This differential expression of cytokine receptors can be used to identify and distinguish a large number of functionally distinct T_reg_ populations and is the major focus of this review.

## Are There Antigen Specific T_reg_?

Acquired or induced immune tolerance is antigen specific, as shown in allograft (6–8, 11) and autoimmune tolerance (47, 48). In autoimmunity induced tolerance is epitope specific (47, 48). The CD4^+^ T cells that transfer transplant tolerance are alloantigen specific (6–8, 11). Antigen specific T_reg_, not polyclonal nT_reg_, are needed to prevent autoimmunity including myelin basic protein induced EAE (49), type I diabetes (50–52), gastritis (53), and peptide specific T_reg_ control EAE induced by that peptide (54).

Animals with tolerance to an antigen or allograft do not have a major increase in CD4^+^CD25^+^ T cells, which remain at ratios of approximately 1:10 to CD4^+^CD25^−^ T cells (55, 56). As these antigen specific T_reg_ represent a fraction of the CD4^+^CD25^+^ T cells, they suppress the immune response at ratios well below 1:10, whereas nT_reg_ are required at non-physiological ratios of 1:1 to suppress *in vivo* (57) and *in vitro* (58, 59). Ratios of 1:1 have only transiently been achieved with IL-2/IL-2 mAb complexes where they can suppress pancreatic islet allograft rejection and autoimmunity (60). It has recently been appreciated that the number of nT_reg_ that need to be produced for transfer to induce tolerance is impossibly large (61). Thus generation of antigen specific T_reg_ from nT_reg_ that suppress at ratios of <1:10 in an antigen specific manner would be highly desirable. We have described how such antigen specific T_reg_ can be generated from naïve nT_reg_ *in vitro* with 3–4 days of culture (46).

## Is There More than One Antigen Specific Subset of T_reg_?

There is ample evidence that the pathways for activation of nT_reg_ and iT_reg_ are multiple and complex, producing antigen specific T_reg_ that control different subpopulations of effector CD4^+^ T cells, including Th1, Th2, Th17, and Tfh cells. The generation of antigen specific T_reg_ from either naïve nT_reg_ or effector lineage CD4^+^CD25^−^ T cells, is complex involving activation of antigen specific T cells with antigen in an environment of cytokines that promotes maturation and clonal expansion of these antigen specific T_reg_. The cytokines that induce these lineages differ and relate to the environment present at the location of activation.

Our hypotheses are that: (i) every phase of the immune response is regulated to some degree, and that T_reg_ are integral to control of all immune responses. (ii) All normal immune response, both *in vivo* and *in vitro*, are associated with activation of a CD4^+^ T_reg_ response. (iii) T_reg_ activation is driven by the cytokines present, including those produced by activated effector T cells. (iv) The more advanced or aggressive the immune response, the more potent the T_reg_ that are generated by the cytokines produced, to control the response. We propose there are several levels of regulation by different functional subclasses of CD4^+^ T_reg_ that are induced and activated by the ambient cytokines. Some of these separate T_reg_ lineages and types are described in Table 1.

**Table 1:** **Subclasses of CD4^+^ T cells with regulatory function**.

**(A) PRESENT TO CONTROL AUTOIMMUNITY IN NORMAL HOSTS**
**nT_reg_** produced in thymus and released into periphery, prevent activation of destructive autoimmune responses. Absence of nT_reg_ due to neonatal thymectomy (19), lack of IL-2, CD25, or FoxP3 (223) leads to widespread autoimmunity. Expression of CTLA4 is required for function of nT_reg_ (224). These cells will control low level immune responses, and suppress at a ratio of 1:1 with more aggressive immune responses (58) including fully allogeneic responses (57, 59). They inhibit antigen presenting cells by direct contact and act in peripheral lymphoid tissues not at sites of inflammation
**Induced T_reg_** generated when antigen is presented in a non-inflammatory environment, when TGF-β is present in the absence of activated antigen presenting cells and inflammatory cytokines such as IL-1β and IL-6. This produces additional T_reg_, that are antigen specific to prevent induction of autoimmune response, in situations where self antigen is released due to non-inflammatory tissue injury such as trauma, ischemia, or chemical injury of tissue as well as in normal tissue re-modeling and failed or incomplete apoptosis, reviewed (225). In these circumstances TGF-β produced to promote repair of tissue also induces iT_reg_ to prevent unwanted and unnecessary autoimmune responses. Their survival is ephemeral if there is repair of tissue, but they may be further activated if inflammation supervenes
**Th3 and Tr1 cells** produced in mucosal sites, in response to antigens that penetrate the mucosa. There is abundant IL-10 and IL-10 family of cytokines, as well as TGF-β at these sites, that promotes tolerance induction to normal mucosal flora and oral antigens to prevent local and unwanted immune responses and inflammation that would disrupt the mucosal integrity. They are essential to the preservation of mucosal integrity and act by production of TGF-β and IL-10 that in turn promotes induction of more Th1 and Tr1
**(B) PRESENT AFTER ACTIVATION OF AN IMMUNE RESPONSE TO A SPECIFIC ANTIGEN**
**Antigen Activation of nT_reg_** by inflammatory immune responses with cytokines produced early after activation of effector CD4^+^ T cells. The best described is the effects of high concentrations of IL-2, inducing expansion of nT_reg_ in the presence of a specific antigen. IL-4 also can induce activation of antigen specific T_reg_ from nT_reg_. Th1 and Th2 responses induce expansion of antigen specific T_reg_, respectively called Ts1 and Ts2 cells, that control responses other that that of the inducing response. This contributes to polarization to one response, for example Th2 cytokine activated nT_reg_ inhibit Th1 and Th17 responses
**Activation of antigen specific activated nT_reg_** by cytokines produced late in an ongoing immune response. This induces the T_reg_ to express cytokines and transcription factors of the activated Th cells, so the T_reg_ become Th-like and express the transcription factor and late cytokines of that Th lineage
**Conversion of activated effector cells to regulatory cells**
(i) Activated T_reg_ infecting activated T cells, via IL-35/IL-10 (226) or surface TGF-β (227 ) to a regulatory T cell phenotype and function
(ii) Persistent activation of effector lineage induces them to produce IL-10 and dampen their own response as was described some 20 years ago (228–230)

## Why are Antigen Specific T_reg_ Hard to Identify?

A key unanswered question is the relationship of naïve non-antigen specific T_reg_ generally described as nT_reg_, to antigen specific T_reg_. In particular whether antigen specific T_reg_ are derived from nT_reg_ or a product of activation of effector lineage CD4^+^CD25^−^ T cells, now known as iT_reg_ (62). Whilst some conclude that antigen specific T_reg_ are mainly iT_reg_, this review will examine the pathways by which nT_reg_ can be activated to antigen specific T_reg_, raising the possibility that activation of nT_reg_ may be the dominant source of antigen specific T_reg_.

Our thesis is based on our findings that antigen specific T_reg_ die *in vitro* and *in vivo*, unless stimulated by specific antigen and cytokines produced by activated effector cells during immune response to the antigen (10–12). This makes identification of antigen specific T_reg_ very difficult, unless they are re-exposed to specific antigen and the cytokines they depend upon. Further, antigen specific T_reg_ do not require IL-2, and in fact may be killed by IL-2 (12). Thus most current protocols for the *ex vivo* expansion of nT_reg_ will not promote antigen specific T_reg_.

## Antigen Specific T_reg_ Express Cell Surface Markers of Activated T Cells

Activated T_reg_ express different cells surface markers to nT_reg_. As examples nT_reg_ express CD45RA and are CD44^lo^, whereas activated T_reg_ express markers of memory cells, being CD45RO^+^ and CD44^hi^. CD45RC is a marker of an activated T_reg_ (11). Class II MHC is only expressed by activated T_reg_, and is a marker of these cells in man (63) and rats (11) but not in mice. nT_reg_ express CD62L and re-circulate from blood to lymph, whereas activated T_reg_ lose expression of CD62L and migrate through peripheral tissue not through lymphoid tissues in murine (64, 65) and humans (66). In naïve CD4^+^CD25^+^ T_reg_, CD62L^+^ not CD62L^−^ T_reg_ suppress GVHD (67, 68). Expression of CCR4 and CCR7, which facilitate migration to lymphoid tissues are expressed by nT_reg_ but not antigen activated T_reg_ (69). Activated T_reg_ migrate to sites of inflammation and express E/P selection (70) and chemokine receptors (65, 71) that will direct them to the site of inflammation that they are programed to control (18). Thus, T_reg_ effective against Th1 responses express CXCR3 (72), those effective against Th2 express CCR8 (73), those for Th17 express CCR6 (74), and those for Tfh express CXCR5 (75).

### Activation of T_reg_ to express transcription factors and cytokines of Th lineages, making Th-like T_reg_ that suppress the relevant Th response

Cytokines normally associated with induction and function of Th1, Th2, Th17, and Tfh CD4^+^ T cells are now found to play a key role in the induction, maintenance, and function of activated T_reg_. Transcription factors that were considered the master regulators of Th responses, play an essential role in activated T_reg_ function, including T-bet the Th1 transcription factor (76), GATA3 the Th2 transcription factor (77), and RORγt the Th17 transcription factor (78). There is plasticity in Th cell lineages, in that various lineages can at time express transcription factors and cytokines not classical for the lineage (79). Epigenetic modification of transcription factor genes and miRNA expression contribute to stability of a lineage, but this can be broken, discussed by O’Shea and Paul (79). CD4^+^CD25^+^FoxP3^+^ T_reg_ can express Th effector lineage transcription factors, together with FoxP3, thereby retaining T_reg_ capacity.

## Activation of T_reg_ in Association with Th1 Responses

In our studies, culture of nT_reg_ with a specific alloantigen and either IL-2 or IL-4 induce antigen specific T_reg_ within 3–4 days of culture (46). They suppress the capacity of naïve CD4^+^ T cells to proliferate *in vitro* to specific donor at 1:32–64 and to effect rejection of specific donor grafts at 1:10 (46), whereas nT_reg_ only fully suppress at 1:1, both *in vivo* and *in vitro* (46, 57, 59). In an autoimmune model, antigen specific T_reg_ were also induced *in vitro* by culture with specific autoantigen and IL-2 that prevented disease *in vivo* (unpublished results). No other Th1 or Th2 cytokines promote proliferation of nT_reg_, including IFN-γ, IL-12p70, IL-12p40, IL-5, IL-13, nor did TGF-β, and IL-10 (46).

With CD4^+^CD25^+^ T cells from animals with tolerance to a fully allogeneic graft, we found that IL-2 or IL-4 induces proliferation to self, specific donor, and third party alloantigen. Proliferation of these T_reg_ to specific donor, and not to self or third party, is promoted by IFN-γ, IL-12p70, and IL-5, but not TGF-β, IL-12p40, IL-10, or IL-13 (Hall et al., unpublished data). These cytokines became candidates for the promotion of survival of alloantigen specific CD4^+^ T_reg_ *in vitro*, where we had not yet identified the specific cytokines involved (12). We had shown that antibody blocking IFN-γ (12) IL-5 and TGF-β (55) does not prevent transfer and maintenance of tolerance by CD4^+^ T cells from tolerant animals, however. Polyclonal activation of nT_reg_ was induced by self antigen and IL-2 or IL-4, and with an antigen proliferation of nT_reg_ induced by IL-2 or IL-4 was further increased (46).

**Figure 1:**
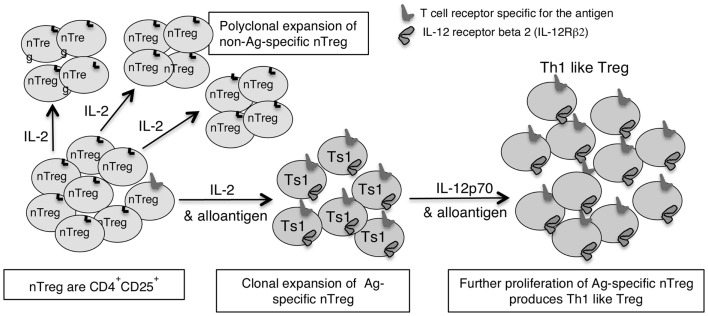
**Shows how IL-2 without TCR engagement with specific Ag induces polyclonal expansion of nT_reg_**. If antigen is present a minority population of nT_reg_ that have TCR specific for antigen are activated to Ts1 by IL-2 and their specific antigen. Ts1 cells express IFNGR, IL-12Rβ2, IL-5, and FoxP3 but not IFN-γ, T-bet, or IL-2. The second step of activation of nT_reg_ converts Ag specific Ts1 to Th1-like T_reg_ and requires specific antigen and either IL-12 or IFN-γ in the absence of IL-2. The Ts1 are antigen specific T_reg_ that continue to express FoxP3, CD25, and CD4, but also express IFNGR, IL-12Rβ2, T-bet, and IFN-γ. Ts1 cells have increased potency over nT_reg_ of at least 10-fold that is antigen specific. Th1-like T_reg_ have 100- to 1000-fold increased suppressor potency over nT_reg_.

**Figure 2:**
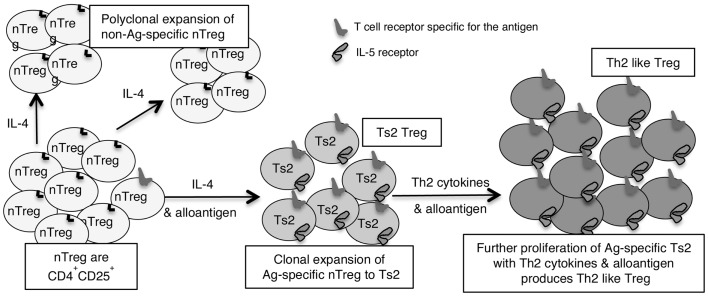
**Shows how IL-4 without TCR engagement with specific Ag induces polyclonal expansion of nT_reg_**. If antigen is present a minority population of nT_reg_ that have TCR specific for antigen are activated to Ts2 by IL-4. Ts2 cells express IL-5Rα, IFN-γ, and FoxP3 but not IL-5, IFNGR, IL-12Rβ2, GATA3, T-bet, or IL-2. Ts2 cells have increased potency over nT_reg_ of at least 10-fold that is antigen specific. The second step of activation of nT_reg_ converts antigen specific Ts2 to Th2-like T_reg_ and requires specific antigen and Th2 cytokines, probably IL-5. Th2-like T_reg_ express IRF4 with FoxP3 and Th2 cytokines IL-4 and IL-5.

This led us to examine if there are two pathways for activation of antigen specific T_reg_, one promoted by Th1 cytokines and the other by Th2 cytokines (46). We identified separate pathways for Th1 and Th2, and called the early Th1 activated T_reg_, Ts1 cells, and the early Th2 activated T_reg_, Ts2. The characteristics of these cells are summarized in Table 2, which also shows that Ts1 and Ts2 cells are an intermediate step in the activation of antigen specific T_reg_, and that they can be further activated by late Th1 and Th2 cytokines to more potent Th1-like T_reg_ (Figure 1) or Th2-like T_reg_ (Figure 2).

**Table 2:** **Summarizes the differences in Th1 and Th2 activated Ag specific T_reg_ and nT_reg_**.

Gene expression	nT_reg_	Subclasses of Ag specific CD4^+^CD25^+^ T regulatory cells			
		Th1 induced		Th2 induced	
		Ts1	Th1-like T_reg_	Ts2	Th2-like T_reg_
IFNGR	+	+++	++	−	?
IL-12Rβ2	−	++	+++	−	?
IL-5Rα	−	−	−	+++	?
IL-4Rα	−	++	?	++	?
IL-2	−	−	−	−	−
IFN-γ	+/++	−	+++	+++	?
IL-4	++	++	?	++	++
IL-5	−	++	++	−	++
IL-10	++	++	?	++	+
TGF-β	++	++	++	++	?
FoxP3	+++	+++	+++	+++	+++
T-bet	−	−	++	−	?
GATA3	−	−	−	−	?
IRF4	?	?	?	?	+++
STAT1	−	?	++	?	?
Chemokine ligand	CCR4	?	CXCR3	?	CCR8
Receptors	CCR7				

### IL-2 and antigen activation of nT_reg_

In cultures of naïve CD4^+^CD25^+^FoxP3^+^ T_reg_ with allo or autoantigen and IL-2, we found that within 2–4 days there was a change in phenotype of the cells, see Table 2. Their expression of mRNA for interferon-γ receptor (IFNGR) increases (46) and the receptor for IL-12p70 (IL-12Rβ2) is induced, whereas the receptor for IL-5 (IL-5Rα) is not induced. There is also enhanced expression of mRNA for IL-5 and reduced expression of IFN-γ. Other cytokine expression remains unchanged, with no IL-2, and similar expression of IL-4, IL-10, and TGF-β to that of fresh naïve nT_reg_. Foxp3 expression is maintained in the majority of cells, and there is no induction of T-bet or GATA3. These changes are not observed when nT_reg_ are cultured with IL-2 and self antigen, suggesting these changes occur related to activation of antigen specific T_reg_. We called these cells Ts1 (46).

Ts1 cells are more potent than nT_reg_ in suppression *in vitro*, as they fully suppress naïve CD4^+^ T cells proliferation in MLC at 1:32–1:64 (46), whereas nT_reg_ only fully suppress MLC at 1:1 or greater (59). Evidence that antigen specific T_reg_ are activated is that Ts1 cells suppress specific donor allograft rejection mediated by naïve CD4^+^ T cells at a ratio of 1:10 (46), whereas naïve nT_reg_ only suppress rejection at 1:1 (57), and Ts1 cells do not suppress third party rejection at 1:10 (46). The animals where Ts1 suppressed rejection, develop tolerance to the allograft and after 150 days have CD4^+^CD25^+^FoxP3^+^ T cells that expressed IFNGR and IL-5, consistent with these Ts1 cells retaining their phenotype over a long period and being key to the maintenance of tolerance.

In other hosts with transplant tolerance, we identified CD4^+^CD25^+^FoxP3^+^ T cells that expressed IFNGR and IL-5, that *in vitro* respond to specific donor and not third party when IFN-γ is present (Hall et al., unpublished data). Further the capacity of tolerant CD4^+^ T cells to transfer tolerance is maintained *in vitro* by culture with specific donor and IFN-γ not IL-2 (Nomura et al., unpublished data). We suggest that these Ts1 maintain alloantigen specific tolerance but are dependent on production of IFN-γ by Th1 cells.

In an autoimmune model we have also generated antigen specific Ts1 cells *in vitro* by culture of nT_reg_ with IL-2 and autoantigen. These Ts1 are induced to express IFNGR and IL-5, and suppressed the autoimmunity in an antigen specific manner (Tran et al., unpublished data).

We suggest induction of Ts1 cells is a key step in induction of antigen specific tolerance to Th1 responses. Ts1 would be promoted by the IFN-γ produced by an ongoing Th1 response, after they stop producing IL-2, which is an early Th1 cytokine. Ts1 cells may in part account for the paradoxical anti-inflammatory effects of IFN-γ, reviewed (80, 81).

### IFN-γ and activation of antigen specific T_reg_

IFN-γ is better known as a pro-inflammatory cytokine, but also has well described effects that control immune responses. IFN-γ directly inhibits Th2 and Th17 cell development, but promotes Th1 responses, including B cell isotype switching, macrophage activation, and cytotoxic T cell development. Activation of the Th1 lineage depends upon IFN-γ activating STAT1, which induces the Th1 transcription factor T-bet, which in turn regulates IFN-γ production by Th1 cells. Once CD4^+^ T cells are activated to a Th1 lineage, they cannot be converted to a T_reg_ lineage (82). IFN-γ is key to CD8^+^ T cell mediated rejection (83, 84) and to allograft vasculopathy (85–87). IFN-γ also activates macrophages to M1 cells and promotes Ig switching to a complement fixing isotypes. IFN-γ promotes MHC class I and II expression on inflamed tissues such a during rejection (88). By induction of MHC class I, IFN-γ protects allografts from CD8^+^ T perforin/granzyme mediated rejection (84, 89–91).

IFN-γ can limit inflammation (92). IFNGR deficient mice have increased severity and reduced recovery from EAE (93, 94). IFN-γ induces iNOS to produce NO, which limits inflammation (95–98). IFN-γ treatment inhibits GVHD (99). CD8^+^ T cells deficient in IFN-γ mediate more severe GVHD, indicating IFN-γ produced by these cells inhibits the CD8^+^ T cell response by inhibiting proliferation and promoting cell death. CD8^+^CD45R^lo^ T cells induced to express IFN-γ, in turn induced indoleamine 2,3-dioxygenase (IDO), and accounts for promotion of indefinite allograft survival after blocking the CD40–CD40L interaction (100).

IFN-γ is also important in the generation and function of CD4^+^CD25^+^ T_reg_ that mediate allograft tolerance (101) and prevents immune destruction of tumors (102). *In vitro*, IFN-γ promotes induction of alloantigen specific CD4^+^CD25^+^FoxP3^+^ T_reg_ that prevent rejection (103). This work by Wood’s group in Oxford identifies that naïve CD4^+^ T cell cultured over a period of time in MLC supplemented with IFN-γ, produces antigen specific T_reg_ that can prevent rejection (41, 103–107). Whether IFN-γ induces iT_reg_ or expands nT_reg_ or a combination of both is unclear. One possibility is that nT_reg_ are initially activated by IL-2 produced by the activated CD4^+^CD25^−^ T cells to induce antigen specific Ts1 cells, that in turn are activated by IFN-γ to expand and maintain the antigen specific T_reg_ (as shown in Figure 1), while a variety of factors such as IFN-γ induction of NO or IDO by antigen presenting cells or IFN-γ promotion of antigen specific T_reg_ may reduce the growth of the effector lineage. IFN-γ inhibits induction of iT_reg_ from CD4^+^ T cells (82), whereas other report IFN-γ is key to induction of CD4^+^CD25^−^ T cells to iT_reg_ that suppress autoimmunity in IFN-γ deficient mice (108).

### Th1-like T_reg_

Th1-like T_reg_ were first described in 2004 associated with a polarizing Th1 response to ovalbumin (109). Ovalbumin specific T_reg_ are induced from CD4^+^CD25^−^ T cells by mature CD8α^+^ DC that produced both IL-12 and IL-10 that are required to induce Th1-like T_reg_ (109). These Th1-like T_reg_ express both FoxP3 and the Th1 transcription factor T-bet, as well as ICOS, IFN-γ, and IL-10. The Th1-like T_reg_ suppressed Th1 inflammation *in vivo* (109). In cancer, Th1-like T_reg_ expressing FoxP3, helios, T-bet, IFN-γ, CXCR3 suppress Th1 responses and are associated with infiltrating Th1 effector cells, probably impairing tumor immunity (110). T-bet expression is required for full T_reg_ function, as T-bet deficient nT_reg_ do not fully control autoimmunity in FoxP3 deficient scurfy mice (72).

T_reg_ induced by activation with a specific alloantigen become FoxP3^+^IFN-γ^+^ and suppress in an antigen specific manner (111). Human iT_reg_ that express T-bet, IFN-γ, and CXCR3 are CD4^+^CD25^+^FoxP3^+^ T cells and suppress (112). Th1-like IFN-γ producing CD4^+^CD25^+^FoxP3^+^ T_reg_ are present in the blood of multiple sclerosis and renal transplant patients during active immune responses (113, 114). Th1-like T_reg_ can be induced by IFN-γ, IL-12, or IL-27 and each may be a separate lineage, albeit they all express FoxP3, T-bet, STAT1, IFN-γ but not IL-2.

### IFN-γ promotes Th1-like T_reg_

Thymus derived nT_reg_ activated in a Th1 environment initially by IL-2, can be further activated by IFN-γ inducing STAT1 to promote expression of the Th1 transcription factor T-bet (115). Absence of STAT1 results in impaired CD4^+^CD25^+^ T_reg_ development and increases host susceptibility to autoimmunity (115). These STAT1/T-bet/FoxP3^+^ T_reg_ control Th1 responses and express CXCR3, which promotes their migration to sites of Th1 inflammation (72). IFN-γ induces T-bet^+^CXCR3^+^ T_reg_ that inhibit Th1 responses in the periphery (116). Collectively these studies confirm IFN-γ can act on T_reg_ to increase their effectiveness in controlling Th1 responses, albeit excessive activation by IFN-γ can reduce their suppressive capacity and may convert them to effector Th1 cells.

### IL-12 promotes Th1-like T_reg_

IL-12p70 is a hetero-dimer composed of p35 and p40 that is produced by APC not T cells (117). IL-12 is a pro-inflammatory cytokine that enhances Th1 (76, 118), cytotoxic CD8^+^T (119), and NK (120) cell responses by increasing IFN-γ (121).

IL-12p70 acts by binding to a high affinity receptor, which is a hetero-dimer of IL-12Rβ1 and IL-12Rβ2 (122), which when activated by IL-12p70 induces STAT4 and T-bet to stabilize the Th1 phenotype and IFN-γ production (123, 124). Resting T cells do not express high affinity IL-12Rβ2 (117), but both chains are up-regulated by TCR and CD28 stimulation, as well as by IL-2 and IFN-γ. IL-4 and IL-10 decrease expression of IL-12Rβ2.

Because IL-12p70 promotes induction of Th1 and cytotoxic T cell responses, it was predicted to amplify rejection and GVHD (125). Paradoxically, treatment with one dose of IL-12p70 at the time of bone marrow transfer inhibits fully allogeneic GVHD (126). Prevention of GVHD by IL-12p70 is dependent on donor IFN-γ (127) acting via Fas to inhibit donor T cell expansion (128). IL-12p70 treatment delays allograft rejection (98) and inhibits autoimmunity including uveitis (129) and EAE (130). The protective effects of IL-12p70 are associated with induction of IFN-γ and iNOS (129). Blocking IFN-γ or iNOS with L-NIL prevents IL-12p70 prolonging graft rejection (98). In other models IL-12 promotes autoimmunity (131–133).

IL-12p35^−/−^ (134), IL-12Rβ2^−/−^ (135), IFN-γ^−*/*−^ (136), and IFNGR^−/−^ (94) mice are more prone to type I diabetes and have reduced numbers of CD4^+^CD25^+^FoxP3^+^ T_reg_ that are less suppressive *in vitro* (137). Some T_reg_ express the IL-12Rβ2 (137). In a situation of an uncontrolled Th1 response, IL-12p70 induces T_reg_ to express T-bet and with high IL-12p70 levels these T_reg_ produce IFN-γ (138). These changes only occur when there is limited IL-2 (138).

In our studies, nT_reg_ cultured with IL-2 and alloantigen (Ts1) expressed IL-12Rβ2 and proliferated with IL-12p70. Ts1 cells activated by specific antigen and IL-12p70 in the absence of IL-2 had greater capacity to suppress alloimmune responses *in vitro* at 1:1000 and *in vivo* at 1:100 (Verma et al., unpublished data). Further, these Ts1 cells cultured with IL-12p70 in the absence of IL-2, expressed mRNA for T-bet and IFN-γ. They continued to express CD25, FoxP3, and mRNA for IFNGR and IL-12Rβ2. Ts1 cultured with IL-2 and IL-12p70 did not express mRNA for T-bet or IFN-γ. The concept of how Th1 cytokines induce Ts1 cells that are activated to a specific antigen to express IFNGR and IL-12Rβ2, and the effects of IFN-γ and IL-12p70 on their further expansion of Ts1 to Th1-like T_reg_ is illustrated in Figure 1.

Many of the anti-inflammatory effects of IL-12p70 are attributed to increased production of IFN-γ that in turn induces iNOS to produce NO (98) but this was not required for Th1-like T_reg_ development *in vitro*. Our results suggested that Ts1 cells, express IL-12Rβ2, and that IL-12p70 directly promotes T_reg_ proliferation and maturation of Ts1 to more potent Th1-like T_reg_ similar to that described by others (72, 138).

### IL-27 promotes Th1-like T_reg_

IL-27 is a member of the IL-12 family of hetero-dimers, that was thought to promote Th1 responses (139). A subset of CD4^+^CD25^+^ T_reg_ express IL-27Rα (140) a receptor required to control excess inflammation during infection (141). IL-27 inhibits Th1, Th2, and Th17 by direct inhibition of cells and induction of T effectors to produce IL-10 (142, 143). IL-27 promotes T-bet and CXCR3 expression in T_reg_ at mucosa sites (116). IL-27 produces specialized T_reg_ that control immunity at sites of inflammation and these T_reg_ appear to express IL-27 as well as IL-27Rα (116). For IL-27 iT_reg_ to function, they must express IFNGR1 and IL-10 (116). The IL-27 induced Th1-like T_reg_ express different genes to Th1-like T_reg_ induced by IFN-γ (116).

IL-27 via the STAT1 pathway, promotes FoxP3 expression by STAT1 binding to the FoxP3 promoter region in iT_reg_ (144).

## Activation of T_reg_ in Association with Th2 Responses

Dominance of Th2 responses (145–148) and Th2 cytokines IL-4 (148–150), IL-10 (151), and IL-13 (152), can protect against autoimmunity, but their effects are variable. Th2 cytokine expression is associated with prolongation of allograft survival in some models (153–158), including neonatal (159–161), and irradiation (162, 163) induced tolerance, but not in all models (164). Th2 cells transfer protection against chronic rejection (165) but do not directly mediate tolerance (166).

### IL-4 effects on nT_reg_ and iT_reg_

IL-4 is key to the induction of Th2 responses by binding to the IL-4Rα and common gamma chain and inducing STAT6 signaling (167) which is required for GATA3 and Th2 cell induction (168). IL-4 makes Th2 cells resistant to T_reg_ (169).

IL-4 also induces STAT6 in T_reg_ and stabilizes expression of FoxP3 (169). GATA3 is essential for full expression of FoxP3 by nT_reg_ and binds to a conserved element of the FoxP3 locus to enhance transcription of FoxP3 (170). GATA3 expression is required to maintain FoxP3 expression in nT_reg_ (77). GATA3 binds to the CNS2 site of the *Foxp3* promoter site as well as the Th2 locus, whereas in Th2 cells it only binds to the Th2 locus (77). This induction of GATA3 in nT_reg_ is not via the IL-4/STAT6 pathway (171), whereas induction of GATA3 via the IL-4/STAT6 pathway in nT_reg_ and iT_reg_ (172) suppresses FoxP3 expression by binding to the FoxP3 promoter region (172).

GATA3 is induced in nT_reg_ during inflammation, and sustains FoxP3 expression (171) especially in T_reg_ at sites of low grade inflammation such as mucosa and skin. Absence of GATA3 in T_reg_ results in a spontaneous inflammatory disorder and defective nT_reg_ that gain a Th17 phenotype (77). Th1 polarizing conditions down regulate GATA3 in Th2 and T_reg_ cells (77). GATA3 induced in nT_reg_ in early inflammation inhibits induction of polarizing factors and generation of effector T cells from nT_reg_ (171). This early induction of GATA3, is dependent upon IL-2 as it is enhanced by IL-2/anti-IL-2 mAb complexes and is absent in IL-2 deficient mice (171).

TGF-β inhibits T-bet expression (173) and GATA3 expression (174) in CD4^+^ T cells reducing Th1 and Th2 cell expansion, thereby favoring FoxP3 expression and iT_reg_ development. On the other hand GATA3 inhibits FoxP3 expression in iT_reg_ activated from CD4^+^ T cells by TGF-β (77) and diverts the cells to an IL-9 producing effector CD4^+^ T cell (175, 176). Thus IL-4 may promote nT_reg_, but inhibit induction of iT_reg_ by promoting GATA3 induction, that down regulates FoxP3 expression. GATA3 is not expressed by RORγt or T-bet expressing T_reg_, nor by Th17 and Th1 cells (171).

IL-4 in culture prevents apoptosis of mice nT_reg_ (177), but IL-4 does not induce proliferation of nT_reg_ only inducing proliferation of CD4^+^CD45RB^hi^CD25^−^T cells (177). IL-4 enhances the capacity of nT_reg_ to suppress IFN-γ induction in CD4^+^CD25^−^ T cells (177). Others found IL-4 induces nT_reg_ proliferation (178) and expression of CD25, FoxP3, and IL-4Rα (169, 177). In cultures, IL-4 induces proliferation of both CD4^+^CD25^+^ and CD4^+^CD25^−^ T cells but promotes survival of CD4^+^CD25^−^ T cells countering inhibition by nT_reg_ (179).

### IL-4 and antigen activation of nT_reg_

We found IL-4 and antigen in culture induced nT_reg_ to antigen specific T_reg_ (46, 56). This activation induces expression of the specific receptor for IL-5 (IL-5Rα) as well as for IL-4 (IL-4Rα) but not IFNGR or IL-12Rβ2, that we observe in cultures with IL-2 and an antigen (46). We call these antigen and Il-4 activated T_reg_, Ts2 cells (46). They continue to express FoxP3, but do not express GATA3, T-bet, or IL-2 (46). Ts2 cells features are summarized in Table 2. Ts2 cells have less expression of IL-5, enhanced expression of IFN-γ, and no change in expression of IL-4, IL-13, TGF-β, or IL-10 (46) (Table 2). These changes are not observed when nT_reg_ were cultured with IL-4 and self antigen, suggesting they are due to activation of antigen specific T_reg_ (see Figure 2).

Ts2 cells have increased potency of suppression *in vitro* as they fully suppressed naïve CD4^+^ T cells proliferation in MLC at 1:32 (46), whereas nT_reg_ only fully suppress MLC at 1:1 or greater (59). Evidence that Ts2 cells are antigen specific T_reg_ is that Ts2 cells suppress specific donor allograft rejection mediated by naïve CD4^+^ T cells at a ratio of 1:10 (46), whereas naïve nT_reg_ only suppress rejection at 1:1 (57). Ts2 cells do not suppress third party rejection at 1:10 demonstrating the Ts2 cells are antigen specific (46). The animals restored with Ts2 cells to suppress rejection develop tolerance to the allograft and after 150 days have CD4^+^CD25^+^FoxP3^+^ T cells that expressed IL-5Rα and IFN-γ. These tolerant T_reg_ proliferate in culture to specific donor, but not to self or third party alloantigen, if IL-5 is present (46). This is consistent with these alloantigen specific T_reg_ retaining their phenotype over a long period and IL-5 being key to the maintenance of tolerance mediated by antigen specific CD4^+^CD25^+^FoxP3^+^ T_reg_.

In other hosts with transplant tolerance, we have identified CD4^+^CD25^+^FoxP3^+^ Ts2 cells that expressed IL-5Rα and IFN-γ, that *in vitro* responded to specific donor and not third party when IL-5 was present (unpublished). Alloantigen with IL-5, but not IL-4, promoted *in vitro* survival of transplant tolerance transferring alloantigen specific CD4^+^ T cells (Plain et al., unpublished data). We suggest that these Ts2 cells maintain alloantigen specific tolerance, albeit animals with tolerance can have both antigen specific Ts1 and Ts2 cells.

In an autoimmune model, we have also generated antigen specific Ts2 cells *in vitro* by culture of nT_reg_ with IL-4 and autoantigen. These Ts2 cells are induced to express IL-5Rα and IFN-γ, not IFNGR, and IL-12Rβ2 (56).

Human CD4^+^CD25^+^CD127^lo^Foxp3^+^ T cells cultured with antigen and IL-4 express IL-5Rα consistent with a human Ts2 cell (56).

We concluded that induction of Ts2 cells is a key step in induction of antigen specific tolerance to Th2 responses. Ts2 would be promoted by the IL-5 produced by an ongoing Th2 response, after the Th2 cells stop producing IL-4, an early Th2 cytokine.

### IL-5 and antigen activation of nT_reg_

As IL-5Rα is not expressed by any other T cells subtype, and is mainly expressed by eosinophils and mast cells, and in rodents B cells, we proposed that IL-5 may be a therapy that could promote immune tolerance by activation and expansion of antigen specific Ts2 (56). Treatment with IL-5 delays neonatal heart allograft rejection and inhibit Th1 cytokine induction (180).

In an autoimmune demyelination model, IL-5 therapy given before disease onset prevents clinical disease and nerve demyelination. IL-5 therapy given after onset of disease, reduces clinical severity of disease and the number of demyelination nerves (56). This is associated with an increase in CD4^+^CD25^+^ T_reg_ and these T_reg_ express IL-5Rα. Further responses of these hosts T_reg_ to the immunizing antigen are enhanced by adding IL-5 to cultures (56). The effect of IL-5 are abrogated by treatment with monoclonal antibodies to deplete CD25^+^ cells or to block IL-4, confirming that the nT_reg_ of the host are activated by antigen and exposure to IL-4 produced in the immune response to the autoantigen (56). The IL-5 therapy promotes expansion of the IL-5Rα expressing antigen specific Ts2 cells (56). IL-5 therapy markedly reduces tissue inflammation and expression of mRNA for the Th1 cytokines IL-2 and TNF-α as well as the Th17 associated cytokine IL-17A. The Th2 cytokines IL-4 and IL-5 are not suppressed (56). This suggests that Ts2 cells may selectively suppress Th1 and Th17 responses, while sparing the Th2 response that produces the IL-4 and IL-5 required for the induction and expansion of Ts2 cells. Thus these Ts2 cells contribute to polarization of Th2 responses by suppressing Th1 and Th17 cells.

Human CD4^+^CD25^+^CD127^lo^FoxP3^+^ T_reg_ cultured with antigen and IL-4, but not IL-2, express IL-5Rα, suggesting IL-5 may promote these antigen specific T_reg_ (56).

### Th2-like T_reg_

Th2-like T_reg_ express the transcription factor Interferon regulatory factor-4 (IFR4) to control Th2 responses (73). IRF4 also promotes Th2 and Th17 (181) responses. IRF4 binds to the promoter region of FoxP3 and induces T_reg_ to express IL-4 and IL-5 (73). Thus induction of IRF4 results in a Th2-like T_reg_. Antigen specific Th2-like T_reg_ are induced in Th2 responses by IL-10 and ICOS/ICOS ligand interaction and secrete IL-10 and some IL-4 but not IL-13 (182). ICOS expressed on T_reg_ promotes their expansion in sites of inflammation during parasitic infestation, whereas in lymphoid tissues ICOS promotes Th2 responses not T_reg_ expansion (183).

During parasitic infestations, CD4^+^CD25^+^ T_reg_ develop in parallel with the Th2 polarization and regulate the size of the immune response (184). These Th2 iT_reg_ inhibit Th1 responses, thereby facilitating Th2 polarization (185, 186). The early immune response to parasites is markedly controlled by T_reg_ (187). Persistence of parasitic infestation is due to CD4^+^CD25^+^ T_reg_ (188, 189) and these hosts have expanded CD4^+^CD25^+^FoxP3^+^ T_reg_ populations (190).

Chronic infestation with parasites is associated with dominance of T_reg_, which suppress Th1 and Th2 responses against the parasite (191, 192). Animals who fail to eliminate parasites have protective CCR8^+^CD4^+^CD25^+^ T_reg_ producing IL-10 that regulates Th2 response (193). Transfer of CD4^+^CD25^−^ T cells confer some protection against infestation, while transfer of activated CD4^+^CD25^+^FoxP3^+^CD103^+^ T_reg_ impairs parasite clearance with greater effect than nT_reg_ (194).

Animals with parasitic infections and an active Th2 response are resistant to the induction of autoimmunity (195, 196) through the effects of TGF-β (197) and have delayed allograft rejection (198–200). This suggests the Th2 milieu and possibly Th2 activated T_reg_ protect these animal from Th1 and Th17 responses (201).

Multiple sclerosis patients with eosinophilia from parasitic infestation have markedly reduced episodes of relapses and new MRI lesions in brain associated with increased CD4^+^CD25^+^ T_reg_ (202). Treatment of parasitic infestations leads to increased relapses and progression of multiple sclerosis with a reduction in T_reg_ (203). Trials of therapeutic parasitic infestation are underway in inflammatory bowel disease (204) and MS (205). As parasitic infestation is associated with Th2 responses and production of IL-5, that induces eosinophilia, one possibility is that this IL-5 promotes antigen specific Ts2 cells to control autoimmunity.

A plausible hypothesis is that the evolution of the immune system was with persistent parasitic infestations and Th2 responses that inhibit innate and Th1/Th17 immunity (206). There is an increasing incidence of autoimmunity in the Western World where the parasitic infestation rate has markedly declined (206). Parasites induction of immune responses that promote T_reg_, possibly by production of IL-5, may also explain the reduced incidence of autoimmunity in populations that live closer to the equator and have poorer hygiene (206).

Our hypothesis is that persistent Th2 responses releasing IL-5 may through a by-stander effect promote expansion of activated antigen specific IL-5Rα^+^ T_reg_ generated to new non-parasite antigens. We demonstrated that IL-5 was an essential growth factor for nT_reg_ activated by IL-4 and these Ts2 cell reduce autoimmune injury (56). We propose that one of the beneficial effects of parasites may be the high IL-5 level produced by a chronic Th2 response, promotes IL-5Rα expressing antigen specific Ts2 cells to control autoimmunity and allograft rejection.

## Activation of T_reg_ in Association with Th17 Responses

### Th17-like T_reg_

T regulatory cells expressing both FoxP3 and IL-17 occur in mice and man (78, 207). IL-17 producing T_reg_ are produced in the periphery not the thymus (78). STAT3, a transcription factor required for Th17 induction, is also required in T_reg_ for induction and maintenance of FoxP3 expression induced by CD28 co-stimulation to produce iT_reg_ (208). Specific deletion of STAT3 in T_reg_ results in a fatal Th17 meditated colitis (209). It is proposed that STAT3 and FoxP3 together coordinate expression of a set of genes that specifically regulate Th17 effector T cells (209). STAT3 induces the receptors for IL-10, and for the pro-inflammatory cytokines IL-6 and IL-23 on Th17 cells and presumably on T_reg_ associated with Th17 responses. IL-27 inhibits T_reg_ via STAT3 (210). IL-10 at the site of inflammation can promote activated FoxP3^+^ T_reg_ and FoxP3^−^ Tr1 (211) and can directly inhibit Th17 and Th17/Th1 cells at the site of inflammation in colitis (212). This suggests that IL-10R is expressed by Th17, Th1/Th17 cells, as well as Th17-like T_reg_ that suppress Th17.

Human peripheral blood and lymphoid tissue contain CD4^+^FoxP3^+^ T_reg_ that express CCR6 and when activated produce IL-17. They express both FoxP3 and RORγt (78). These CD4^+^CD25^+^FoxP3^+^ cells, that produce IL-17, strongly inhibit CD4^+^ T cell proliferation, and could be cloned (78). Naïve CD4^+^FoxP3^+^CCR6^−^T_reg_ that have their TCR stimulated in the presence of IL-1β, IL-2, IL-21, and IL-23 differentiate into IL-17 producing T_reg_ (78). Human T_reg_ that secrete IL-17A express the Th17 transcription factor RORγt (213). Both naive and memory T_reg_ suppress Th17 cells and inhibit their production of IL-17 and IL-22, as well as their expression of CXCL8 (214).

CD4^+^CD25^+^FoxP3^+^ T_reg_ expressing IL-17, that acquire IL-1R1 can be converted to Th17 cells by IL-1β (215). This group suggested the preferred route of induction of Th17 in man may be via activation of nT_reg_ with lineage differentiating factors, such as activated APC, IL-1β, TGF-β, and IL-23 as well as IL-2 (74). They propose a new role for nT_reg_ as precursors of Th17 effector cells. IL-2 therapy triggers conversion of Th17 producing FoxP3^+^ T_reg_ to Th17 cells that do not express FoxP3 (216). The Th17 effectors, that no longer suppress, do not express FoxP3 or IL-1R1, but express CCR6; similar to a smaller population of T_reg_ that express FoxP3 and IL-17 (74).

IL-21 synergizes with IL-2 to promote activation of effector CD4^+^ and CD8^+^ T cells but inhibits induction of iT_reg_ when combined with IL-2 and TGF-β (217). Thus, there is evidence for activated T_reg_ and iT_reg_ being induced to suppress Th17 responses that use induction pathways, in part, shared with Th17 cells.

## Activation of T_reg_ in Association with Tfh Responses

Tfh-like T_reg_ are specialized T_reg_ that control germinal center expansion and autoimmune responses that are found in primary B cell follicles. These CD4^+^CD25^+^FoxP3^+^ T cells migrate to the T-B border areas of secondary lymphoid tissues, where they suppress Tfh dependent antibody responses by inhibiting both B cells and T cells (218, 219) These cells are CD4^+^CD25^+^FoxP3^+^ T cells that share transcription factors and cell surface phenotype with Tfh cells, including expression of the Tfh chemokine receptor CXCR5 (75, 219) and PD1 which is expressed by Tfh (75). The development of Tfh-like T_reg_ is similar to Tfh cell development as it depends upon expression of the transcription factor Bcl-6 (75). Bcl-6 is a transcription factor that promotes Tfh and represses other Th lineages. They also express Blimp-1, which is repressed in B cells and Tfh that express Bcl-6 (75). Bcl-6 is a transcriptional repressor that promotes Tfh but represses other Th lineages. Bcl-6^−/−^T_reg_ are selectively impaired at controlling Th2 responses, but not Th1 and Th17 responses, as Bcl-6 suppresses GATA3 and Th2 (220).

Both Tfh and Tfh-like T_reg_ depend upon SAP, CD28, and B cells for their activation (75). Similar to Tfh cell induction, the Tfh-like T_reg_ are induced by IL-21 and IL-6 and produce IL-21 with STAT3 expression. Tfh-like T_reg_ are derived from nT_reg_ and are not iT_reg_ (75). Tfh-like T_reg_ prevent over expansion of germinal centers and mediate tolerance in B cell responses.

## Conclusion

This review sets out the evidence that nT_reg_ are activated by cytokines released by the activation of CD4^+^CD25^−^ T cells in all immune responses. It describes how the responsiveness of antigen activated nT_reg_ changes during the immune response. Initially nT_reg_ are activated by early cytokines such as IL-2 in Th1 and IL-4 in Th2 responses. With persistent active immune responses, the cytokines produced change. In late Th1 responses IFN-γ and IL-12p70, not IL-2 is produced, and these late Th1 cytokines further expand and activate IL-2 and antigen activated Ts1 cells. In late Th2 responses IL-5 and IL-13 are produced not IL-4. In late Th2 response IL-5 promotes IL-4 and antigen activated Ts2 cells.

Excessive amounts of these cytokines can further induce antigen specific T_reg_ to express the transcription factor of the dominant inflammatory response, so that in Th1 responses T-bet and STAT1 are induced to Th1-like T_reg_ that produce IFN-γ. In Th2 responses T_reg_ express IRF4 and produce IL-5 and IL-4 to become Th2-like T_reg_. In Th17 responses activated T_reg_ express RORγt and IL-17A to become Th17-like T_reg_, whereas in Tfh responses, T_reg_ express Bcl-6, and IL-21 to become Tfh-like T_reg_. Each step of activation is associated with an increase in potency to suppress of the activated T_reg_, so that they can suppress at ratios of 1:10–1:1000, whereas nT_reg_ only fully suppress at 1:1. These subsets are identifiable by expression of chemokine ligands, CXCR3 in Th1 responses, CCR8 in Th2 responses, CCR6 in Th17 responses, and CXCR5 in Tfh responses. Highly potent antigen specific T_reg_, with the potential to migrate to sites of tissue inflammation to control active destructive immune responses, has far reaching potential in therapy for allograft rejection, control of GVHD, and autoimmunity.

These activated T_reg_ include antigen specific T_reg_ and require specific antigenic stimulation and the relevant cytokines to promote their survival. The requirement for specific antigen and a restricted cytokine milieu makes study of these cells *in vitro* very difficult, unless the correct environment is created to promote their survival. Further, the expansion of enriched nT_reg_ by repeated culture with IL-2 over more than a week, only expands nT_reg_ and probably selects against antigen specific T_reg_ as the cytokines required to sustain antigen specific T_reg_ are absent and IL-2 prevents induction of Th1-like T_reg_.

It is now appreciated that the number of nT_reg_ to control GVHD, graft rejection, or autoimmunity is impossibly large, as they need to be present at ratios of 1:1 or greater (221). Understanding the pathways for selective activation of antigen specific T_reg_ from nT_reg_ will allow growth of more potent T_reg_ that suppress in a specific manner with smaller numbers of cells. This may be achieved by first culturing nT_reg_ with IL-2 or IL-4, then with other cytokines, respectively IFN-γ or IL-12 and IL-5. The effector mechanisms of each subset or activated T_reg_ also needs resolutions, as there are many effector mechanism other than inhibition of APC with CTLA4 and production of IL-10 and TGF-β, as reviewed (222).

## Conflict of Interest Statement

Bruce M. Hall and Suzanne J. Hodgkinson hold patents related to T regulatory cells. The other co-authors declare that the research was conducted in the absence of any commercial or financial relationships that could be construed as a potential conflict of interest.
